# Pediatric food allergies in primary care: the GPs perspective

**DOI:** 10.1186/2045-7022-5-S3-P75

**Published:** 2015-03-30

**Authors:** Aideen Byrne, Orla O'Dwyer, Sinead Ramjit, Mary Kate Byrne, Adam Boissoneault, Brendan Browne

**Affiliations:** 1 Our Lady's Children's Hospital Crumlin, Dublin, Ireland; 2 The School of Medicine, Trinity College, Dublin, Ireland

## Background

Childhood food allergy (FA) prevalence continues to increase. Skilled primary care involvement is essential for effective management.

## Objective

The study sought to obtain feedback from GPs in Ireland regarding their approach to paediatric FA, and their access to training and expert advice.

## Methods

An online questionnaire was composed and distributed to GPs, recruited through a database, across the Republic of Ireland. A quota of 100 complete questionnaire responses was set. All analyses were performed on SPSS and MS Excel statistical software.

## Results

66% of GPs report that they always or almost always take a focussed FA history for patients presenting with urticaria. Significantly more histories were taken for urticaria than for feeding problems (p=0.007), eczema (p=0.025) or asthma (p<0.001). Self-reported confidence in diagnosing FA correlated directly and significantly with confidence in interpreting specific IgE results (p<0.001). Perception of confidence in diagnosis and management of FA was significantly greater in those who have had training in allergy management in the last 5 years (p<0.001) and those who are aware of local guidelines (p=0.004). GPs with higher confidence scores in interpreting specific IgE testing results had a better understanding of who to contact for specialist advice(p=0.009) and more often sought advice (p=0.036). 99% of GPs surveyed expressed interest in learning more about paediatric FA.

## Conclusions

This study demonstrated variability in the confidence of GPs to deal with paediatric food allergies. It also revealed an appetite for further training and suggests targeted education is effective in increasing confidence amongst GPs in diagnosing and managing FA.

